# Psychosocial interventions for children exposed to traumatic events in low- and middle-income countries: study protocol of an individual patient data meta-analysis

**DOI:** 10.1186/2046-4053-3-34

**Published:** 2014-04-11

**Authors:** Marianna Purgato, Alden L Gross, Mark JD Jordans, Joop TVM de Jong, Corrado Barbui, Wietse Tol

**Affiliations:** 1 World Health Organization Collaborating Centre for Research and Training in Mental Health and Service Evaluation, Section of Psychiatry, University of Verona, Policlinico G.B. Rossi, Piazzale L.A. Scuro 10, 37134 Verona, Italy; 2 Department of Mental Health, Johns Hopkins Bloomberg School of Public Health, Hampton House, N. Broadway 624, 21205 Baltimore, USA; 3 Department of Epidemiology, Johns Hopkins Bloomberg School of Public Health, Monument St. 2024 E, 21231 Baltimore, USA; 4 Department of Research & Development, HealthNet TPO, Lizzy Ansinghstraat 163, 1072RG Amsterdam, The Netherlands; 5 Institute of Psychiatry, Kings College, De Crespigny Park 16, SE5 8AF London, UK; 6 Amsterdam Institute of Social Science Research, University of Amsterdam, OZ Achterburgwal 185, 1012 DK Amsterdam, The Netherlands

**Keywords:** Children, Individual patient data meta-analysis, Mental health, Low- and middle-income countries, Randomized controlled trials

## Abstract

**Background:**

The burden of mental health and psychosocial problems in children exposed to traumatic events in humanitarian settings in low- and middle-income countries is substantial. An increasing number of randomized studies has shown promising effects of psychosocial interventions, but this evidence has shown complexity with regard to setting, conflict-phase, gender, and age. These complex findings raise the need of a detailed evaluation of the specific factors which influence size and direction of intervention effects.

Individual patient data meta-analysis is a specific type of meta-analysis that allows the collection of exact information at an individual patient level, and to examine whether intervention and socio-demographic characteristics, trauma-related variables, environmental conditions, and social support may act as moderators and mediators of intervention effect.

The aim of the present study is to carry out an individual patient data meta-analysis using data from all available randomized controlled trials (either published or unpublished) comparing psychosocial intervention with waiting list or no intervention arms in children exposed to traumatic events living in low- and middle-income countries.

**Methods/Design:**

All randomized trials comparing selective preventive psychosocial intervention versus waiting list or no treatment conditions in children (0–18 years) living in low- and middle-income countries will be included. Studies will be identified in accordance with the Preferred Reporting Items for Systematic reviews and Meta-Analyses guidelines. There will be no restrictions on publication type, status, language, or date of publication. The primary outcome measures will be psychological symptoms (post-traumatic stress disorder, anxiety, depression). Secondary outcomes will be positive mental health outcomes (coping methods, social support, self-esteem), and function impairment.

**Discussion:**

We are expecting that some variables, like socio-demographic characteristics, trauma-related variables, environmental conditions, and social support will act as moderators/mediators of intervention effect. The investigation of the role of these factors on the intervention effects will help in the appropriate selection, development, implementation, and dissemination of evidence-based programs in low- and middle-income countries.

**Trial registration:**

This protocol has been registered with the International Prospective Register of Systematic Reviews (PROSPERO) (registration number: CRD42013006960).

## Background

The burden of mental health and psychosocial problems in children exposed to traumatic events in humanitarian settings in low- and middle-income countries (LMICs) is substantial [1], although precise estimation of this burden is challenging. The term ‘humanitarian settings’ refers to a broad range of emergency situations, including armed conflicts, political violence, and natural or industrial disasters. Such humanitarian crises predominantly affect populations in LMICs [2]. Most research on child mental health in humanitarian settings in LMICs has focused on the identification of post-traumatic stress disorder (PTSD) and depressive symptoms [3], with gaps in evidence on other types of mental health conditions, protective factors, functioning, disability, and social, physical, educational, marital, and occupational outcomes [4-6]. Additionally, qualitative research has pointed to the importance of contextual risk factors for child mental health in areas of armed conflict, including undermined social support networks, socio-economic adversity, and ongoing lack of access to basic needs [7]. Despite the large body of literature on the relevance and impact of traumatic events on children’s mental health, and in spite of an increasing number of rigorous studies showing promising effects of psychosocial interventions, the evidence supporting the efficacy of these interventions has shown complex results. A range of evaluations of school-based interventions have found promising improvements in child mental health on both psychological symptoms and positive and function impairment outcomes, but generally for specific sub-groups only. In addition, in two cluster randomized trials in Burundi and Sri Lanka, both positive and negative effects of interventions for subgroups were identified [6,8-10].

These complex findings raise the need of a detailed evaluation of the specific factors which influence the size and direction of intervention effects. Research in this area is ongoing. For example, a cluster randomized trial on a school-based mental health intervention with conflict-affected children in Burundi analyzed the role of some variables – such as gender, age, level of trauma exposure, household size, family connectedness, and others – as moderators and mediators of the effect of interventions [6]. This study reported intervention benefits for the outcome ‘hope’ (the sense of hope in terms of being able to generate solutions and being able to implement these in problem situations) in younger children and in children with low levels of past exposure to traumatic events. For children living in larger households an improvement in the depressive symptoms outcomes and function impairment was found. Moreover, children living with both parents showed benefits in the depressive symptom outcomes and PTSD. In both Burundi and Sri Lanka, subgroups of children in waitlist arms showed better trajectories on some outcomes (i.e., for girls on PTSD symptoms in Sri Lanka, and for displaced children on hope and function impairment in Burundi). The authors explained this in socio-ecological terms. In a setting with ongoing adversity, children in more stable settings (lower past trauma exposure, larger households, living with both parents) benefited from the intervention. Similarly, a randomized controlled trial of the same school-based intervention in Sri Lanka found stronger improvements in children with lower levels of ongoing conflict-related stressors. In addition, gender and age were found to moderate intervention effects.

In contrast, a randomized trial carried out in conflict-affected areas of Indonesia, but with seemingly less impact of conflict on family and community functioning, showed a larger improvement of function impairment for children living in smaller households and receiving social support from members outside the family. Significant associations were also found between gender and PTSD symptoms, with girls showing larger improvement than boys in this outcome [9,10]. Overall, these findings point to a potential contextual ‘tipping point’ for the impact of school-based interventions in settings of armed conflict, or to a differential benefit for specific subgroups of youth. That is, in settings where children live in relatively supportive family environments, school-based interventions may show overall positive effects. In contrast, in situations where children live in more vulnerable situations (e.g., ongoing conflict-related stressors, severe strains on family functioning, dire poverty) school-based interventions may have benefits for some children, but may have unfavorable effects on others. In the latter situation, school-based preventive interventions with more homogenous groups and a stepped care approach may be advised (e.g., selected by gender, age, past trauma exposure, ongoing stressors, and displacement status) in order to maximize benefits and reduce chances for doing harm. Based on these findings, further work in Burundi has explored the selection of an appropriate parenting intervention for particularly vulnerable families as a starting point for intervention [11,12], whereas a study showed the benefit of individual counseling for more seriously afflicted children in Sudan [13].

Even though these trials/studies represent a step toward improved understanding of the mediator and moderator role of intervention effects, further analyses are required to shed light on the processes and conditions that influence effects of psychosocial interventions for children affected by armed conflict.

By means of individual patient data (IPD) meta-analysis, a specific type of meta-analysis that allows the collection of exact information at an individual patient level, it is possible to examine whether intervention and socio-demographic characteristics, trauma-related variables, environmental conditions, and social support may act as moderators and mediators of intervention effect. IPD meta-analysis has been shown to be the gold standard to re-analyze patient-level data offering greater statistical power to carry out informative subgroup analyses and to explore the mediator and moderator roles of some selected variables [14,15]. In IPD meta-analysis, the original research data for each participant of each included study are sought directly from the researchers responsible for that study [16,17]. The advantages of using raw data are that more exact information is available on individual patient level about subgroup status, and it offers the opportunity to recode variables (i.e., making them more comparable between trials), to include all randomized patients and to improve the overall follow-up rates.

The aim of the present study is to carry out an IPD meta-analysis using data on file from all available randomized controlled trials (RCTs) (either published or unpublished) comparing preventive psychosocial intervention with waiting list or no intervention in children exposed to traumatic events living in LMICs. A comprehensive analysis will help to understand why some children benefit from some interventions more than others, and then to aid in appropriate selection, development, dissemination and implementation of evidence-based interventions in LMICs.

### Objective

To investigate the effectiveness of preventive psychosocial interventions for children exposed to traumatic events in LMICs by means of a comprehensive systematic review of all RCTs conducted on this topic.

In order to examine whether clinical and socio-demographic characteristics, trauma exposure-related variables, environmental conditions, and social support may act as moderators and mediators of intervention effect, an IPD meta-analysis will be carried out. The systematic review and the IPD meta-analysis will be conducted according to the methodology recommended by the Cochrane Collaboration [16].

### Study hypotheses

#### Hypothesis 1

Psychosocial preventive interventions will be moderated by individual variables (for example gender, age, displacement status, exposure to traumatic events).

#### Hypothesis 2

Psychosocial preventive interventions will be mediated by social support (for example household size, family connectedness) and coping methods.

We defined moderator and mediator as follows:

Moderator: a variable that affects the direction and/or strength of the relation between an independent or predictor variable and a dependent or criterion variable.

Mediator: an event that occurs after treatment onset but precedes outcome, is associated with independent variable, and has an interactive effect on treatment outcome [10,18].

## Methods/Design

### Types of studies

Any studies that allocated participants or clusters of participants by a random method and included a no-intervention standard care or wait list condition will be included. The study selection process will be reported in accordance with the Preferred Reporting Items for Systematic reviews and Meta-Analyses (PRISMA) guidelines [19]. There will be no restrictions on publication type, status, language, or date.

### Types of participants and settings

Children of both sexes (aged 0–18 years) who have been exposed to a traumatic event in the context of a humanitarian crisis, and residing in a LMIC. The review will not be restricted to participants meeting specific psychiatric diagnostic categories (as we are interested in selective preventive interventions), but will include participants who have been exposed to traumatic events related to humanitarian crises (e.g., displacement, exposure to violence, or major losses). Traumatic events and humanitarian settings will be defined according to the criteria used in a recent systematic review and meta-analysis of mental health and psychosocial support interventions in LMICs [20]. Humanitarian settings will include countries that have had a humanitarian crisis, including any natural or technological disasters and political violence (armed conflicts and wars). LMICs will be selected using the World Bank criteria [21]. Interventions delivered in any setting will be accepted, including healthcare facilities, refugee camps, schools, communities, survivors’ homes, and detention facilities.

### Types of interventions

Interventions will include selective preventive psychosocial interventions compared to no intervention or waiting list. Selective preventive interventions will be defined according to the ecodevelopmental model of prevention proposed by Weisz [22]. According to this model, selective prevention targets subgroups of children with specific risk factors (for example children with psychological distress); who are not already experiencing a disorder [22].

### Types of outcome measures

#### Primary outcomes

##### Mental health

PTSD symptoms (measured with the Child Posttraumatic Symptom Scale [23], Clinician Administered PTSD Scale for Children and Adolescents [24] (or with any other commonly used rating scales).

Anxiety (measured with the Screen for Anxiety Related Emotional Disorders (SCARED-5) [25], or with any other commonly used rating scales).

Depression (measured with the Children Depression Rating Scale [26], Depression Self-Rating Scale (DSRS) [27], or with any other commonly used rating scales).

#### Secondary outcomes

##### Resilience

Hope (measured with the Children’s Hope Scale (CHS) [28] or with any other commonly used rating scales).

Coping (measured with the Kidcope [29] or with any other commonly used rating scales).

Social support (measured with the Social Support Inventory Scheme [30] or with any other commonly used rating scales).

Functioning (measured with the Child Function Impairment Measure [31] or with any other commonly used rating scales).

Loss to follow-up.

### Search methods for identification of studies

The search method will start with an update of the search strategy performed by Tol et al. in a systematic review of RCTs on mental health and psychosocial interventions in LMIC humanitarian settings in 2011 [20]. The Cochrane Collaboration Trials Registers of the Developmental Psychosocial and Learning Problems Group [32], the Depression, Anxiety and Neurosis Group [33], and the Schizophrenia Group [34] will be searched to identify other relevant studies. These registers are compiled by systematic searches of major databases, hand searches, and conference proceedings. Medline, Embase, PsycInfo, CENTRAL, and PILOTS will be searched as well.

These sources will be supplemented by searching reference lists of relevant papers and previous systematic reviews and relevant specialist websites.

Moreover, the following regional databases will be hand searched for additional studies not retrieved in the electronic database and for citations of unpublished reports: AfricaBib databases, African Index Medicus, AFROLIB Database, Biomedicina Croatica, Chinese Medicine Premier, East View Information Service, EurasiaHealth, Hellenic Ph.D. Dissertations Thesis, HERDIN NeON Database, Hrcak, Index Medicus for the Western Pacific, Indian Citation Index, IndMED, InfoMED, IranMedex, KoreaMed, LILACS, Magyar Orvosi Bibliográfia, MedCarib, Medical Bibliography Hippocrates, Medical databases (Russia), Panteleimon, Turk MEDLINE, Türk Tıp Veri Tabanı, and University of Zagreb Medical School Repository.

### Study selection

Material downloaded from electronic sources will include author, institution, or publication journal details. As an initial step, on the basis of titles and abstracts, two reviewers (MP and WT) working independently will select potentially relevant studies. Studies rated as possible candidates by either of the two review authors will be added to a preliminary list and their full texts will be retrieved.

No blinding to the names of authors, institutions, and journal of publication will take place. We will resolve any further disagreements by consensus with a third member of the review team (CB).

### Data collection, transfer, and management

The principal investigator of each included trial will be contacted by email to ask cooperation on the project and to confirm study eligibility. We will prepare a standard template in which we will introduce our research group and the objectives of this work, and we will list the individual and study level information requested from study authors. Attached we will forward the study protocol of this IPD meta-analysis. Principal investigators will be asked to contribute to the IPD meta-analysis with the original datasets. Study level information will include study protocol, published papers, and unpublished or additional material. Individual level information will include socio-demographic and clinical characteristics, primary and secondary outcome measures, date of randomization, and date of follow-up.

All study data will be entered in a computerized password-protected database, only accessed by named study staff and stored by the Department of Mental Health, Johns Hopkins Bloomberg School of Public Health, Baltimore, United States. The computer will be placed in a secure location protected by a key (physical protection). All study data will be used only for the purposes stated in this Study Protocol, and will not be forwarded to third parties.

### Data extraction

The following documents will be requested for each of the included studies:

– Study protocol;

– Study questionnaires;

– Clinical study report (if available);

– List of publications.

We will assign a unique number to each of the included studies. Study level variables will include: author(s), institution or journal of publication, year of publication, country, setting, duration of the study, type of intervention, recruitment period, primary and secondary outcomes, methodological issues. Risk of bias and quality information will additionally be extracted (see below).

For study level information that will not be provided by the investigators, a reviewer (MP) will extract information from the study protocol, if available, and from published paper(s).

We will assign each child a unique identification number and we will create a dataset from each study with one row per child. Individual level information will include:

Unique identification number (code), age, study, country, date of assessment, time;

– Socio-demographic characteristics: family characteristics, caregivers occupation, religion, displacement experience, household size;

– Exposure to traumatic events: past and current exposure;

– Symptoms: PTSD symptoms, depressive symptoms, anxiety symptoms;

– Resilience (hope, coping, social support);

– Functioning;

– Type of psychosocial intervention (duration, frequency, clinical features).

### Assessment of risk of bias

Knowledge of risk of bias is crucial in interpreting the results of individual studies and in identifying differences between studies that may determine a high level of heterogeneity in the results [35]. The risk of bias of each study will be assessed by two reviewers (MP and CB) using the Cochrane Collaboration risk of bias tool [16]. This comprises a judgment and a support for the judgment for each specific feature of the study (sequence generation, allocation concealment, blinding, incomplete outcome data assessment, selective outcome reporting and other source of bias/locally validated diagnostic instrument). The judgment for each entry involves assessing the risk of bias as ‘low risk’, as ‘high risk’, or as ‘unclear risk’, with the last category indicating either lack of information or uncertainty over the potential for bias [36].

### Data analysis

Descriptive statistics will be used to summarize baseline, clinical, and socio-demographic characteristics of participants in each study, and for the pooled sample. We will describe the characteristics of included studies, the number of studies contributing with data to the IPD meta-analysis, and the types of outcomes provided by each study. The number of excluded studies together with the reasons for exclusion will be also reported (following the PRISMA statement). Results tables, graphs, and statistical syntax will be discussed by the review authors during meetings and/or telephone conferences.

The sample size available for each specific outcome will depend on the completeness and quality of the data from the RCTs.

### Individual level analysis

Two general approaches to IPD meta-analysis have been suggested [37,38]. The first is the ‘one-stage analysis’. This combines all the IPD from all studies to perform a single analysis. This can be in a ‘mega-trial’ analysis, where distinctions between studies are ignored and the data are analyzed as if they belong to a single trial. This approach is feasible even if outcome summary scores differ; common items among scales can be used to link outcomes from multiple studies to a common metric in pooled data. The second is the ‘two-stage approach’. Here, studies are analyzed separately, and then summary statistics combined using meta-analysis techniques. In the current IPD meta-analysis, both these approaches will be employed.

For dichotomous outcomes, relative risk (RR) with a 95% confidence interval (CI) will be calculated based on a random effects model, as this takes into account differences between studies [16]. Continuous scores from different outcome scales will be analyzed using weighted or standardized mean differences with a 95% CI. A random-effects-model will be employed, as this takes into account any differences between studies [16]. Assorted graphical tools and examination of heterogeneity (the I^2^ statistic) will be used to investigate the possibility of statistical heterogeneity among studies. We will interpret the I^2^ estimate as indicating the presence of high levels of heterogeneity if it is greater than or equal to 0.50 [39]. Data from included studies will be entered into a funnel plot. Funnel plot will be visually inspected in order to detect the presence of publication bias [16]. We will adjust for confounding variables in regression models of outcomes on predictors. We will select potentially confounding variables using *a priori* assumptions and exploratory data analysis.

We will analyze mediation using structural equations modeling to implement a one-step procedure that simultaneously models relationships between exposure and mediator, mediator and outcome, and exposure and outcome [40]. The test of mediation will be based on a test of the distribution of the product of z-scores for the associations between the exposure and mediator and the mediator and outcome [41]; this approach has been demonstrated in simulation studies to have superior power than other approaches for our particular case [40]. Potential effect modification will be examined by including terms for exposure, suspected moderator, and the interaction between the two, and testing the significance of the interaction.

A sensitivity analysis excluding studies that failed to provide individual-level information will be carried types of exposure to traumatic events;out. Additionally, subgroup analyses will be conducted grouping studies according to:

types of exposure to traumatic events;

levels of family and social support;

displacement;

household size;

setting;

age; and

sex.

### Publications

The Principal Investigator of each included trial, as well as other investigators with a specific interest for this project, will be asked to be co-authors of any publication(s). These investigators will be asked to provide feedback on data analysis and interpretation, as well as on the writing of the publication(s).

### Ethical issues

The present project does not involve primary data collection from humans, as it will be based on secondary analyses of already collected de-identified datasets. Only studies that have received formal approval from an ethical committee and followed acceptable ethical standards will be included in the present work. Further institutional review board approval will be obtained at the Johns Hopkins Bloomberg School of Public Health. Additionally, national and international regulations on patient privacy will be followed.

## Discussion

This systematic review and IPD meta-analysis will focus on the effects of preventive psychosocial interventions for children exposed to traumatic events in LMIC.

Some variables, like socio-demographic characteristics, trauma-related variables, environmental conditions, and social support have been suggested to act as moderators/mediators of intervention effect, but no formal evaluation has been conducted on this topic so far. Likely, the results of this study may shed light in the field of preventive psychosocial interventions, helping generate a better understanding of the reasons why some children benefit from some interventions more than others. This will in turn facilitate a better matching of interventions with individual and contextual factors in this field.

## Abbreviations

IPD: Individual patient data meta-analysis; LMICs: Low- and middle-income countries; PRISMA: Preferred Reporting Items for Systematic reviews and Meta-Analyses; PTSD: Post-traumatic stress disorder; RCTs: Randomized controlled trials.

## Competing interests

No commercial sponsors are involved in this project. Dr. Marianna Purgato was partially funded by the COOPERINT grant for internationalization of research, sponsored by the University of Verona. During the preparation of this paper Dr. Marianna Purgato was acknowledged a three-year fellowship from the European Commission (Marie Curie International Outgoing Fellowship) to conduct this project. The authors declare that they have no competing interests.

## Authors’ contribution

MP, WT, and CB drafted the protocol. AG supervised the statistical analysis. AG, MJ, and JdJ commented and refined the manuscript in preparation for submission. All authors approved the final version to be published.
